# Proteomic Workflows for Biomarker Identification Using Mass Spectrometry — Technical and Statistical Considerations during Initial Discovery

**DOI:** 10.3390/proteomes1020109

**Published:** 2013-08-27

**Authors:** Dennis J. Orton, Alan A. Doucette

**Affiliations:** 1 Department of Pathology, 11th Floor Tupper Medical Building, Room 11B, Dalhousie University, Halifax, NS B3H 4R2, Canada; E-Mail: dennis.orton@dal.ca; 2 Department of Chemistry, Room 212, Chemistry Building, Dalhousie University, Halifax, NS B3H 4R2, Canada

**Keywords:** biomarker discovery, experimental design, randomization, replication, high dimensional data

## Abstract

Identification of biomarkers capable of differentiating between pathophysiological states of an individual is a laudable goal in the field of proteomics. Protein biomarker discovery generally employs high throughput sample characterization by mass spectrometry (MS), being capable of identifying and quantifying thousands of proteins per sample. While MS-based technologies have rapidly matured, the identification of truly informative biomarkers remains elusive, with only a handful of clinically applicable tests stemming from proteomic workflows. This underlying lack of progress is attributed in large part to erroneous experimental design, biased sample handling, as well as improper statistical analysis of the resulting data. This review will discuss in detail the importance of experimental design and provide some insight into the overall workflow required for biomarker identification experiments. Proper balance between the degree of biological *vs.* technical replication is required for confident biomarker identification.

## 1. Introduction

Having moved into an era of molecular medicine, high throughput ‘omics’ screening methods are being used to decipher informative, disease specific markers promising effective treatment strategies for individualized treatments. With improved gene-based technologies now enabling rapid and cost-effective genome sequencing, researchers are now looking to the proteome as accurate and responsive predictors of the pathophysiological state of an individual. Proteome workflows to identify biomarkers capable of diagnosis [1,2], prognosis [3,4,5], or classification of disease [5,6], primarily center on high-throughput technologies involving mass spectrometry (MS) or microarray technology. These platforms are capable of identifying and profiling the abundance patterns of hundreds to thousands of proteins within a single experiment [7,8], providing a “snapshot” in time of the pathophysiological state of an individual. However, despite the maturing technologies for proteome profiling, identification of clinically relevant biomarkers remains elusive. 

**Figure 1:**
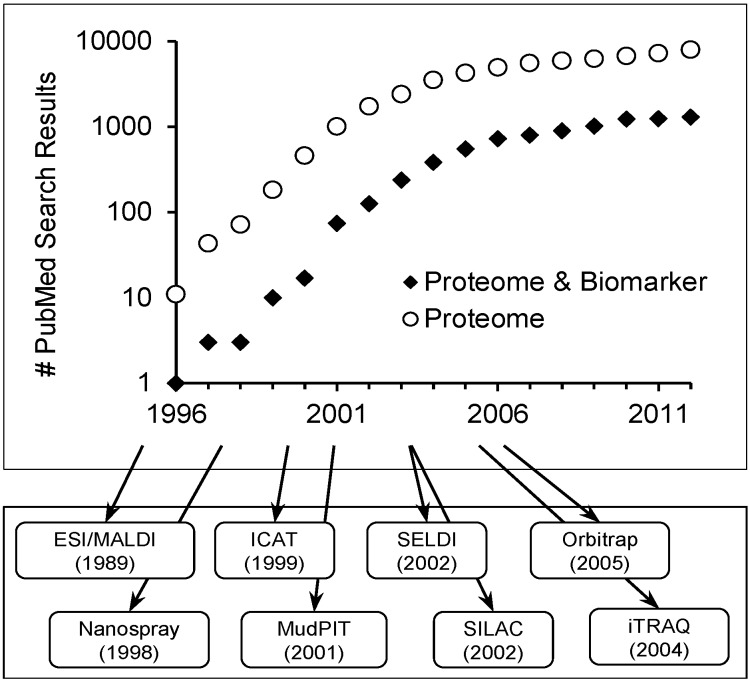
(**Top**) The number of PubMed search results as a function of year; (**Bottom**) The growth in number of publications corresponds directly to the application of numerous technologies and methods [9,10,11,12,13,14,15,16] used to improve throughput and sensitivity.

Since the mid-1990s, research in the area of MS-based proteome analysis is growing exponentially in conjunction with the search for novel disease biomarkers. Figure 1 summarizes the yearly PubMed search results according to the keywords “proteome” or “proteome and biomarker” and highlights the increasing popularity of the field. This explosion of growth has been made possible by technological advances [9,10,11,12,13,14,15,16], permitting quantitative protein analysis in a high throughput manner (Figure 1). A vast number of cell types, diseased tissues, and biological fluids on both clinical samples, as well as *in vitro* or *in vivo* experimentation have been profiled in an effort to bring biomarkers to the clinical setting. Problematically, despite numerous claims of success, no test derived using MS-based proteomic techniques is currently FDA approved. Acknowledging the dynamic complexity of any proteome, this lack of validated biomarkers is ultimately attributed to flaws in experimental design [17,18], the use of biased or inconsistent methodology [19,20], or inadequate statistical analyses [21,22,23]. Innate errors in biomarker discovery experimentation, coupled with irreproducible results in some high- profile cases, have delayed progress and shaken confidence in the field of biomarker research [24,25,26,27]. 

### Scope of Review

This review will discuss the current state of biomarker research, as well as the inherent challenges associated with proteomic technologies for identification of disease biomarkers. It should be noted that a biomarker discovery experiment extends beyond the analytical lab. For example, proper consideration must be given to the number (e.g., multiple patient samples or multiple samples from one patient) and type (e.g., proximal fluid or tissue) of samples to be taken for analysis, the method of sample collection (e.g., anesthetization of the patient or catheterization) and preservation (e.g., storage conditions or inclusion of protease inhibitors). Following discovery of a putative biomarker, a validation phase must be included to determine the efficacy (e.g., sensitivity and specificity) of the biomarker at the clinical level. Methods for validation of biomarkers have been reviewed [28,29] and introduction of a pipeline geared towards bringing proteomic biomarkers into routine clinical use have been suggested [30]. Most importantly, consideration of the points raised in this review must be given during all phases of the biomarker identification process. For example, standardization of collection methods and storage conditions will eliminate bias in the early stages of biomarker discovery, while implementation of the good experimental practices discussed below will reduce bias in data accumulation, allowing the greatest potential for identification of true biomarkers. 

Acknowledging that most reliable biomarker would arise from analysis of the ‘normal’ state of a single individual compared with the ‘diseased’ state of the same individual, this may not be possible. A lack of baseline comparisons, such as in paediatric populations, or knowledge of what sample to analyze, and what to search for make this form of biomarker discovery not feasible for the discovery phase. This review focuses on the fundamentals of experimental design and provides an in-depth analysis of common errors in biomarker discovery experiments that must be addressed prior to execution of the experiment. 

## 2. Sample Description

### 2.1. Characteristics of an Ideal Biomarker

The National Institute of Health defines a biomarker as a *“…characteristic that is objectively measured and evaluated as an indicator of a normal biological process, pathogenic process, or pharmacologic responses to therapeutic intervention”* [31]. With respect to biomarker discovery through genomic or proteomic approaches, the indicating characteristic may be gene(s) or protein(s) that present quantifiable changes in expression across a clinically obtainable sample. What constitutes an ideal biomarker depends heavily on the disease in question, though universal characteristics of the ideal biomarker are summarized in Table 1. 

The stringent requirements for ideal biomarkers presented in Table 1 imply the identification of a single gene or protein biomarker for a given disease to be extremely unlikely. To combat this issue, investigators often turn to panels of genes or proteins which together may provide sufficient information to differentiate test populations based on pathophysiological state [4,5,6,32]. The inclusion of multiple variables in the test population, however, can result in so called ‘overfitting’ of the data, and limit the applicability of the test. Therefore, an efficient biomarker test should also express a property known as generalizability, which allows the test to be applicable to large, diverse populations.

**Table 1:** Universal characteristics of an ideal biomarker.

Characteristic	Description
(1) Non-invasive collection	Expression within a sample obtainable without discomfort to the patient
(2) Readily available	Presentation in an easily obtainable sample that is commonly obtained clinically such as blood or urine
(3) High sensitivity	Allows early detection of disease with little or no overlap between healthy and diseased patients
(4) High specificity	Present in the disease in question, with little or no overlap between comorbid conditions
(5) Rapid response	Changes rapidly in response to treatment
(6) Risk stratification	Provides prognostic information to the clinician, allowing classification of the disease along with diagnosis
(7) Insight to disease	Provides insight into the underlying mechanism of the disease

### 2.2. Sources for Biomarker Identification

The underlying hypothesis of biomarker experimentation is that pathophysiological changes in cells or tissue are reflected through gene or protein expression, preferably in a disease-specific fashion. Biomarker discovery experiments aim to exploit these changes for clinical testing. A range of omics technologies [33,34,35,36,37,38,39] can be applied to investigate changes in *in vitro* or *in vivo* models of disease, or to profile clinical (human) samples to uncover biomarkers. Therefore, careful consideration of the source of biomarker must be given prior to experimentation. The choice of sample generally depends on the method of analysis, but also the disease in question. Table 2 summarizes the innate advantages and disadvantages of various sample sources. A brief discussion of common sample sources and their application to biomarker discovery is provided below. 

**Table 2:** Summary of various sources of biomarkers for discovery platforms.

Source	Advantages	Disadvantages
*In vitro* cell culture	Easy to obtain; no ethics; abundant sample quantity; good for characterizing cell-specific responses	Lack of heterogeneity; may not represent clinically relevant results
Tissue biopsy/core sample	Accessibility to samples stored long term; direct comparison to standard diagnosis; tissue-level representative profiling	Potential for sample degradation; require large validation datasets; invasive sample collection
Urine/blood	Easy to obtain; express representative protein and gene expression of a large number of cell types	Low marker concentration; high sample complexity; technically difficult to detect
Proximal fluid (e.g., Nipple aspirate, bile, prostate, *etc*…)	Representative of the tissue microenvironment over blood/urine; may provide more sensitive results	More difficult to obtain than blood/urine; potentially extremely invasive (e.g., CSF)

***In vitro*** disease models provide a simplified sample source for researchers to elucidate a cell-specific physiological response to disease or treatment. A model culture system has the benefit of limiting several confounding variables which plague clinical samples by controlling test conditions which optimize the model. Cell culture models are common to study the response of a particular cell type to various stimuli [40], as seen for drug toxicity studies, or studying gene knockdown effects. Though useful as early high throughput screening studies, *in vitro* models likely do not reflect the complexity of the disease by excluding the heterogeneity of cells affected by the disease, as well as the heterogeneity of the afflicted population. *In vitro* models employing high throughput ‘-omics’ methods are therefore more commonly reserved for holistic, semi-quantitative assessments of changes in protein or gene expression profiles [41,42]. 

**Tissue biopsies** and core samples are a common source for clinical diagnosis through microscopic evaluation following a number of staining techniques, but can also provide a source for biomarker research. Biopsies can provide researchers with direct access to diseased tissues, and therefore potential biomarkers, making them the most relevant source for biological information during biomarker experimentation. Moreover, because biopsies constitute the traditional route for pathological characterization of many diseases, methods for sample collection, storage, and analysis are now standardized and routine. This offers a potential route to integrate tissue biopsies into biomarker identification platforms. Previously, the fixation and staining processes required for standard pathological assessment prevented coupling to standard proteomic methodologies. More recently, methods have been established which allow direct proteomic analysis of tissue samples following processing (fixation, embedding, sectioning, staining) [43]. As a result, biomarker researcher may access thousands of banked tissue samples collected over extended periods, vastly expanding the number of samples available for analysis [43,44]. Extrapolating from the analysis of tissue banks, a secondary advantage is provided through multi-year follow-up analyses of a given patient, which can evaluate the predictive value of a biomarker. Banked samples are perhaps the only effective strategy to investigate rare diseases, which could take years to compile sufficient samples for true evaluation of effective biomarkers.

Despite the benefits of biopsy analysis, access to the sample by the researcher is restricted - perhaps rightfully so. Obtaining the sample from a patient is not trivial, and has the potential to introduce complications such as infection that could lead to decreased quality of life for the patient. This is especially true if the diseased tissue is present in a sensitive or difficult to access area, which makes collection of appropriate samples a key source of error. Combined with other sources of error which may be introduced by the researcher conducting the analysis, the use of less invasive sources of biomarkers has become a more common strategy for discovery purposes.

**Proximal fluids** are obtained from the extracellular milieu of tissues and contain a wide range of soluble and secreted factors from cells within a tissue microenvironment [45]. Compared to blood, such fluids can provide researchers with a lower-complexity sample in a potentially non-invasive manner, with the added benefit of enriching the sample with proteins with particular relevance to the tissue of interest. Proximal fluids exist in a wide range of biological environments, some of which allowing easy, non-invasive access, while others may be more difficult to access, leading to more stringent ethical considerations for sample collection. Some proximal fluids that have been investigated for biomarkers include cerebrospinal, bronchoalveolar lavage, cervicovaginal, cyst, ascites (abdominal fluid), nipple aspirate, amniotic, and blister, as well as bile, saliva, expressed prostatic secretion and seminal plasma, and pancreatic juice (reviewed by Teng *et al.* [45]). 

**Blood and urine** are by far the most commonly obtained biological samples in a clinical setting and therefore provide an excellent medium for biomarker research. While these samples can be considered proximal fluids themselves, they are generally used as systemic measures, as opposed to tissue-specific indicators (*i.e.*, urine-based pregnancy test). Despite the simplicity of sample collection, biomarker identification from these samples has been especially lacking. Issues stemming from sample collection [46,47,48], storage [49,50], complexity, and protein concentration range [36,51] have been implicated in the lack of progress in the field [52]. Efforts to isolate and correct variables within each of these experimental parameters have begun to allow researchers to draw more informative conclusions from data obtained by these high throughput technologies [19,20].

Proteomic analysis of serum [36] and urine [53] have identified thousands of proteins expressing dynamic ranges up to 12 orders of magnitude, with the 22 most highly abundant proteins in serum making up approximately 99% of the total protein concentration [36]. In an effort to boost the sensitivity of proteomic analyses, a range of pre-fractionation methods have been developed. Gel electrophoresis [54,55], immunodepletion [56,57,58], and various forms of chromatography [59] have been employed to selectively exclude high abundance proteins from analysis, allowing visualization of a broader dynamic range. Despite improved fractionation strategies, the high sample complexity presents researchers with a daunting task when undertaking a biomarker discovery experiment employing these samples. 

**Figure 2:**
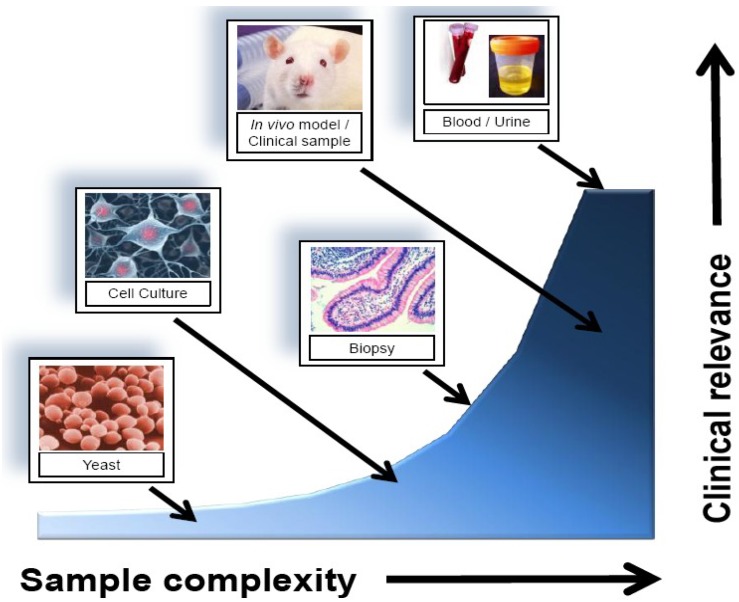
A schematic representation of potential sources of biomarkers. Less complex model systems provide a simpler starting point for biomarker investigation; however, the clinical utility of the analysis improves by transitioning to more complex model systems.

Regardless of source, biological samples contain a complex array of proteins, nucleic acids, and cell signalling molecules, all of which have potential use as disease biomarkers. Figure 2 provides a brief overview of the relative complexity of the samples discussed above. Therefore, choosing the best method for biomarker discovery depends heavily on the disease in question, but also on the source of biomarker to be investigated. 

## 3. Sample Analysis

### 3.1. Biomarker Discovery Experimental Design

Effective experimental design requires formulation of a hypothesis, proper selection of a test group, and allocation of appropriate experimentation to draw unbiased conclusions that support or refute the proposed hypothesis [60]. The proposed hypothesis can be specific in nature, as seen for example when studying the effects of altered gene expression on the proliferation of a cell population. Conversely, as is common to biomarker experimentation, the hypothesis can be much broader in nature and query a large number of genes or proteins within a single experiment. These experiments are said to be ‘discovery-based,’ where the hypothesis simply states that there are some quantifiable differences in the sample caused by a test condition, which distinguish between the test and control groups. High throughput methods may also be used to qualitatively assess global changes in gene or protein expression, such that a specific hypothesis can be formulated and tested in the classical sense [40,61]. No matter the goal of the biomarker experiment, careful planning and immaculate experimental design are of utmost importance. 

A number of biomarker studies have, through a wide range of experimental pitfalls, generated false results. Issues stemming from improper sample population selection [17], sample handling and storage [62], sequential sample analysis [63] and improper sample analysis [25] may have been averted with proper experimental design. Poor design often reveals promising results early in the study, which inevitably cannot be reproducible, or fail to support the hypothesis during subsequent validation. Avoiding bias in experimental design was addressed as early as 1937 when Sir Ronald Fisher proposed construction of an unbiased experimental procedure based on randomization, replication, and blocking [64]. Though fundamental to experimental design, such concepts are often overlooked in proteomic experiments. Additionally, prior to experimentation on ‘real’ clinical samples, it is important to critically assess each stage, or ‘experimental unit’, in the workflow for sources of bias [60,65]. An experimental unit can be the gel on which protein samples are resolved, the isolation or collection of samples, or the method of detection of the sample (*i.e.*, LC-MS analysis). Construction of an unbiased experiment therefore begins with proper understanding of the experimental procedures before running those precious clinical samples. 

**Figure 3:**
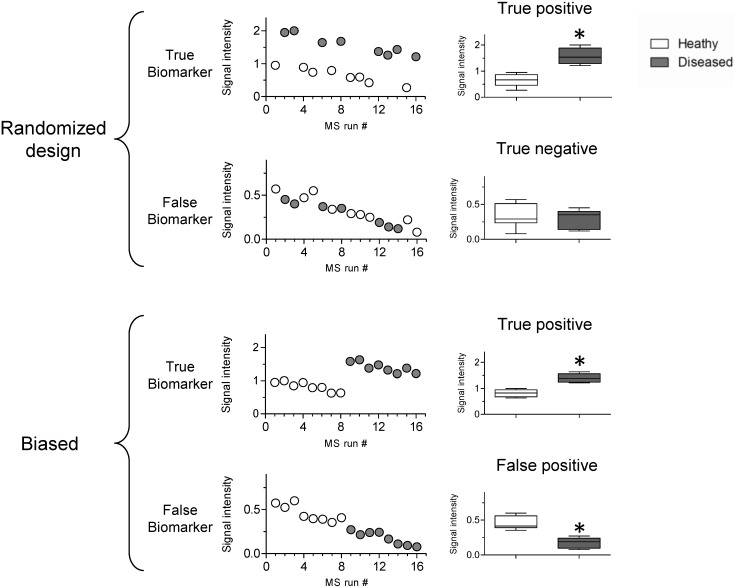
The influence of randomization on quantitative analysis of potential biomarkers. In the figure, successive MS runs were assumed to contribute a 5% decrease in signal intensity. A randomized design allowed proper characterization of the true biomarker while avoiding improper characterization of the false biomarker. In a biased design, the samples were analyzed in an improper grouping, which led to an apparent difference in the observed concentration of the false biomarker.

High throughput biomarker identification studies require adherence to the experimental setup provided by Fisher to prevent bias introduced during analysis. Sequential processing of samples, although potentially easier to execute, is one of the most common, though easily avoided sources of bias. Figure 3 outlines the influence of sequential processing on altering the validity of a biomarker discovery experiment. Here, it is assumed that the mean concentration and standard deviation of a putative biomarker in a test population does not change. However, by ignoring randomization, the confidence in the obtained data can drastically decrease or lead to identification of a false biomarker. The terminology introduced by Fisher as it pertains to high throughput technologies is discussed in greater detail below.

***Randomization*** in experimental design refers to both the collection of samples as well as sample processing and data analysis. Randomization guards against the introduction of uncontrollable variables unknown to the researcher that may affect the accuracy of the data. Changes in response may include factors such as sample collection and storage time, protein extraction and processing, changes in chromatographic separation and instrumental drift. All aspects of the workflow, from sample collection to data interpretation need to be controlled. The effect of randomization is shown in Figure 3. Here it is assumed that a variable in the analysis workflow has introduced drift, corresponding to a 5% relative loss in signal following each successive analysis. A randomized design correctly identifies the true biomarker and sees no statistical difference between the control *vs.* text groups for a ‘false’ biomarker. Without randomization, the 5% signal drift introduced an apparent difference for the false biomarker, invalidating the results of the analysis. Numerous examples of this have been presented in the literature, including the false identification of a biomarker for ovarian cancer, which were later proven to be invalid as the original profiling of diseased *vs.* healthy samples occurred on separate days, yielding the false results [24,66].

***Replication*** allows assessment of biological and technical variability of the biomarker identification workflow while quantifying the range of ‘normal’ *vs*. ‘diseased’ states for potential biomarkers. Technical replication tests the variation within each of the experimental units, such as sample isolation, collection, storage, preparation, or detection. Quantifying the level of technical variation within each experimental unit is essential for determining a threshold above which test groups are statistically different. Knowledge of the variability in the methodology will also assist in prediction the number of biological replicates required to obtain quantitative information [65,67,68]. Biological replication tests the innate inter- or intra-individual variability within a test population. These results require preliminary study of a large population of ‘normal’ samples for estimation of the expected variability within a test population [69]. 

**Figure 4:**
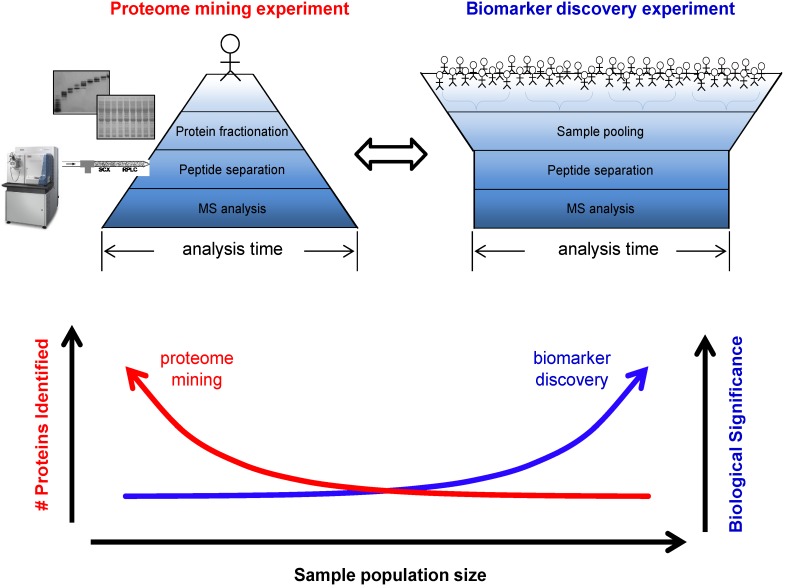
Increasing the level of fractionation greatly improves the number of proteins identified, though longer analysis time is required. Conversely, biomarker experiments require analysis of larger sample sizes to improve the biological significance of the identified proteins. In the discovery phase, biomarker experimentation must find a balance between these extremes. Pooling samples is one method to reduce the analysis time, however will also limit biological relevance.

The majority of variability is assumed to be biological, and so increasing the number of biological replicates will achieve a higher level of confidence in the result [65]. However, more replicates implies longer analysis time. It is well known, given the complexity of the proteome, that a higher level of fractionation (protein or peptide level) allows the researcher to mine the proteome more deeply, increasing the dynamic range of abundance over which proteins are identified. Coupled with the inclusion of technical replicates, the number of individual analysis will expand to the point of requiring unreasonable instrument time to characterize greater than a single proteome. As seen in Figure 4, the biomarker discovery platform cannot be a direct extension of a proteome mining experiment; multiple biological replicates are required for confident biomarker identification. Based on the expected technical and biological variability, it is said that up to thousands of samples are required for definitive conclusions to be drawn for biomarker discovery by high throughput methodologies [65,67,68,70]. Pooling samples from healthy and diseased groups will reduce the sample size while maintaining a high degree of confidence in the data, however, pooling also eliminates the estimation of inter-individual variation within each group, and can mask outliers which can reduce the applicability of the biomarker upon validation [71,72].

***Blocking*** is meant to prevent bias contributed by experimental parameters known to the researcher. Examples of blocking during experimentation include organizing samples based on age, gender or ethnicity, but also on the disease grade or sample origin. Blocking should be applied to insure equal allocation of experimental groups in a randomized trial, allotting equal analysis time of healthy and diseased groups. As an example, a test for obstructive coronary artery disease (CAD) predicts with high confidence the presence of CAD in non-diabetic men, however is ineffective in women, and men with diabetes [1,73,74]. Other factors such as comorbidity or medication also present confounding variables that cannot be corrected by any number of statistical manipulations post acquisition. Blocking is therefore the only way to limit the influences of these variables and implies proper experimental design [60,65].

### 3.2. Sources of Bias

The previous section discussed in detail how employing an unbiased experimental workflow can improve confidence in results by eliminating error during analysis. However, without careful attention to experimental protocols, alternative sources of bias in experimentation can lead to erroneous results. Numerous forms of bias, ranging from sample collection and storage to purification and sample preparation, have contributed to false or low quality biomarker identification in prostate and ovarian cancers [24,25,62,63,75,76]. Detection and correction of these sources of bias is an important aspect of omics research and biomarker discovery [19,20,77,78,79,80]. 

The innate complexity of biological samples can also play a role in erroneous or insensitive results. For example, bottom-up (peptide level) proteomic strategies employing LC-MS generally include a preliminary, protein level form of fractionation ahead of MS analysis, which promotes visualization of a wider dynamic range of proteins. Unfortunately, many strategies employed for fractionation include buffer additives incompatible with LC-MS which must be removed prior to analysis. At best, removal of these compounds can lead to sample loss and decreased sensitivity [81,82,83]. Similarly, depletion of highly abundant proteins or sample purification can introduce unexpected bias in results due to non-specific binding [84,85,86]. Reducing these forms of bias are of utmost importance to the overall experimental workflow, and optimization of each protocol beforehand can lead to greatly improved sensitivity in results. However, dealing with such large volumes of information can provide challenges all their own [22,23,87].

### 3.3. Statistical Analysis of High Dimensional Datasets

Microarray and MS based techniques generate thousands of data points for a single sample [8,53]. As the number of data points per sample approaches hundreds to thousands, the data becomes what is known as ‘high dimensional’ [23]. Traditional statistical methods such as t-tests are commonly used to compare the mean values of two samples to determine statistical significance between them. This method is useful for determining differences between traditional datasets; however these methods break down during analysis of data in high dimensions. A commonly employed *p-*value of a t-test of 95% confidence, implies that 5 times out of 100 the reading is a falsely identified as being significant. Following this logic, conducting a t-test on expression profiles of 10,000 genes or proteins at 95% confidence potentially leads to identification of 500 false positives. Shifting the confidence interval to decrease the number of false positive identifications (referred to as type I error) can theoretically improve confidence in the data; however will lead to a significant increase in false negative identifications (type II error). This confounding statistical problem is known as ‘the curse of dimensionality’ [88].

Obviously, these methods are not ideal for biomarker identification in this fashion, introducing a need for statistical methodology better suited to high dimensional datasets. A number of researchers have published methods in an attempt to circumvent or reduce the effects of the curse of dimensionality [89,90,91]. Such statistical algorithms take advantage of bayesian statistics, hierarchical clustering, or quasi-poisson distribution, and support vector machine (SVM) methods to differentiate between data sets. A comparison of some of these statistical methods was conducted by Leitch *et al.* (2012) [21]. These statistical methods can be used to generate a list of candidate data points contributing to the differences between each group (*i.e.*, diseased or healthy). However, with these statistical approaches, there is a danger of over-fitting the data. Over-fitting occurs as a result of applying a large number of variable data points to a small number of outcomes (*i.e.*, diseased *vs.* healthy). Insuring the quality of data therefore requires a large ‘training’ sample group accompanied by an independent ‘validation’ group. Ransohoff reviewed the terminology [29], and gives examples of sufficient and insufficient data sets for biomarker identification. Because it may not be possible to obtain sufficient samples to construct large training and validation groups, one may conduct cross-validation within the training set by sequential grouping and comparison within the same group [92]. Other methods aiming to eliminate over fitting employing unsupervised statistical methods include principle component analysis [93,94] or hierarchical clustering [95,96]. These methods however shown greater application for classification of disease based on subcellular processes than for biomarker discovery [97,98].

## 4. Conclusions

The field of biomarker discovery using high throughput methodology in the form of microarray chip technology and mass spectrometry is rapidly expanding. Biomarker studies aim to compile gene or protein profiles for effective and decisive disease diagnosis, prognosis, and prediction of effective treatment plans. Early on, the field of biomarker discovery experienced some growing pains in the form of flawed methodologies and inadequate statistical analyses, leading to irreproducible biomarker discoveries. These errors in judgement, as researchers rushed to publish and identify biomarkers unfortunately led to erroneous results. These false claims shook the confidence of researchers and caused a general mistrust of the ability of high throughput technologies to yield informative disease markers. Over the last decade, a large quantity of research has been committed to the critical assessment of sources of bias, statistical analysis, and experimental procedures employed in biomarker discovery studies. This research has provided a better understanding the fundamentals in experimental planning and execution, as well as statistical analysis of such large data sets. 

As technology develops to allow even greater amounts of data to be generated, methods to handle such large datasets must be understood and applied correctly to allow effective conclusions. In response to the need to obtain quantitative information of large data sets, statistical algorithms capable of dealing with them are being developed, and ever-expanding computational power is also allowing a greater number of samples to be analyzed. The future of biomarker research depends on the experiments currently being conducted, and retaining stringent requirements for true identification of biomarkers, with sufficient validation to back claims will boost confidence in the field and allow a greater understanding of the molecular pathophysiology behind a great number of diseases.

## References

[1] Rosenberg S., Elashoff M.R., Beineke P., Daniels S.E., Wingrove J.A., Tingley W.G., Sager P.T., Sehnert A.J., Yau M., Kraus W.E. (2010). Multicenter validation of the diagnostic accuracy of a blood-based gene expression test for assessing obstructive coronary artery disease in nondiabetic patients. Ann. Int. Med..

[2] Dumur C.I., Lyons-Weiler M., Sciulli C., Garrett C.T., Schrijver I., Holley T.K., Rodriguez-Paris J., Pollack J.R., Zehnder J.L., Price M. (2008). Interlaboratory performance of a microarray-based gene expression test to determine tissue of origin in poorly differentiated and undifferentiated cancers. J. Mol. Diagn..

[3] Cronin M., Pho M., Dutta D., Stephans J.C., Shak S., Kiefer M.C., Esteban J.M., Baker J.B. (2004). Measurement of gene expression in archival paraffin-embedded tissues: Development and performance of a 92-gene reverse transcriptase-polymerase chain reaction assay. Am. J. Pathol..

[4] Bedard P.L., Mook S., Piccart-Gebhard M.J., Rutgers E.T., Van’t Veer L.J., Cardoso F. (2009). MammaPrint 70-gene profile quantifies the likelihood of recurrence for early breast cancer. Expert Opin. Med. Diagn..

[5] Deng M.C., Eisen H.J., Mehra M.R., Billingham M., Marboe C.C., Berry G., Kobashigawa J., Johnson F.L., Starling R.C., Murali S. (2006). Noninvasive discrimination of rejection in cardiac allograft recipients using gene expression profiling. Am. J. Transplant..

[6] Ueland F.R., Desimone C.P., Seamon L.G., Miller R.A., Goodrich S., Podzielinski I., Sokoll L., Smith A., van Nagell J.R., Zhang Z. (2011). Effectiveness of a multivariate index assay in the preoperative assessment of ovarian tumors. Obstet. Gynecol..

[7] DeSouza L.V., Siu K.W.M. (2013). Mass spectrometry-based quantification. Clin. Biochem..

[8] De Godoy L.M.F., Olsen J.V., Cox J., Nielsen M.L., Hubner N.C., Fröhlich F., Walther T.C., Mann M. (2008). Comprehensive mass-spectrometry-based proteome quantification of haploid versus diploid yeast. Nature.

[9] Fenn J.B., Mann M., Meng C.K., Wong S.F., Whitehouse C.M. (1989). Electrospray ionization of large for mass spectrometry biomolecules. Science.

[10] Gatlin C.L., Kleemann G.R., Hays L.G., Link A.J., Yates J.R. (1998). Protein identification at the low femtomole level from silver-stained gels using a new fritless electrospray interface for liquid chromatography-microspray and nanospray mass spectrometry. Anal. Biochem..

[11] Gygi S.P., Rist B., Gerber S.A., Turecek F., Gelb M.H., Aebersold R. (1999). Quantitative analysis of complex protein mixtures using isotope-coded affinity tags. Nat. Biotechnol..

[12] Washburn M.P., Wolters D., Yates J.R. (2001). Large-scale analysis of the yeast proteome by multidimensional protein identification technology. Nat. Biotechnol..

[13] Issaq H.J., Veenstra T.D., Conrads T.P., Felschow D. (2002). The SELDI-TOF MS approach to proteomics: Protein profiling and biomarker identification. Biochem. Biophys. Res. Commun..

[14] Ong S.E., Blagoev B., Kratchmarova I., Kristensen D.B., Steen H., Pandey A., Mann M. (2002). Stable isotope labeling by amino acids in cell culture, SILAC, as a simple and accurate approach to expression proteomics. Mol. Cell. Proteomics.

[15] Wiese S., Reidegeld K.A., Meyer H.E., Warscheid B. (2007). Protein labeling by iTRAQ: A new tool for quantitative mass spectrometry in proteome research. Proteomics.

[16] Olsen J.V., de Godoy L.M.F., Li G., Macek B., Mortensen P., Pesch R., Makarov A., Lange O., Horning S., Mann M. (2005). Parts per million mass accuracy on an Orbitrap mass spectrometer via lock mass injection into a C-trap. Mol. Cell. Proteomics.

[17] Reddy M.M., Wilson R., Wilson J., Connell S., Gocke A., Hynan L., German D., Kodadek T. (2011). Identification of candidate IgG biomarkers for Alzheimer’s disease via combinatorial library screening. Cell.

[18] Pepe M.S., Feng Z. (2011). Improving biomarker identification with better designs and reporting. Clin. Chem..

[19] Hu J., Coombes K.R., Morris J.S., Baggerly K.A. (2005). The importance of experimental design in proteomic mass spectrometry experiments: Some cautionary tales. Brief. Funct. Genomic. Proteomics.

[20] Banks R.E., Stanley A.J., Cairns D.A., Barrett J.H., Clarke P., Thompson D., Selby P.J. (2005). Influences of blood sample processing on low-molecular-weight proteome identified by surface-enhanced laser desorption/ionization mass spectrometry. Clin. Chem..

[21] Leitch M.C., Mitra I., Sadygov R.G. (2012). Generalized linear and mixed models for label-free shotgun proteomics. Stat. Interface.

[22] Johnstone I.M., Titterington D.M. (2009). Statistical challenges of high-dimensional data. Philos. Trans. R. Soc. A.

[23] Clarke R., Ressom H.W., Wang A., Xuan J., Liu M.C., Gehan E.A., Wang Y. (2008). The properties of high-dimensional data spaces: Implications for exploring gene and protein expression data. Nat. Rev. Cancer.

[24] Petricoin E.F.I., Ardekani A.M., Hitt B.A., Levine P.J., Fusaro V.A., Steinberg S.M., Mills G.B., Simone C., Fishman D.A., Kohn E.C. (2002). Use of proteomic patterns in serum to identify ovarian cancer. Lancet.

[25] Adam B.L., Qu Y., Davis J.W., Ward M.D., Clements M.A., Cazares L.H., Semmes O.J., Schellhammer P.F., Yasui Y., Feng Z. (2002). Serum protein fingerprinting coupled with a pattern-matching algorithm distinguishes prostate cancer from benign prostate hyperplasia and healthy men. Cancer Res..

[26] McLerran D., Grizzle W.E., Feng Z., Bigbee W.L., Banez L.L., Cazares L.H., Chan D.W., Diaz J., Izbicka E., Kagan J. (2008). Analytical validation of serum proteomic profiling for diagnosis of prostate cancer: Sources of sample bias. Clin. Chem..

[27] McLerran D., Grizzle W.E., Feng Z., Thompson I.M., Bigbee W.L., Cazares L.H., Chan D.W., Dahlgren J., Diaz J., Kagan J. (2008). SELDI-TOF MS whole serum proteomic profiling with IMAC surface does not reliably detect prostate cancer. Clin. Chem..

[28] Rifai N., Gillette M.A., Carr S.A. (2006). Protein biomarker discovery and validation: The long and uncertain path to clinical utility. Nat. Biotechnol..

[29] Ransohoff D.F. (2004). Rules of evidence for cancer molecular-marker discovery and validation. Nat. Rev. Cancer.

[30] Mischak H., Ioannidis J.P., Argiles A., Attwood T.K., Bongcam-Rudloff E., Broenstrup M., Charonis A., Chrousos G.P., Delles C., Dominiczak A. (2012). Implementation of proteomic biomarkers: Making it work. Eur. J. Clin. Invest..

[31] Atkinson A.J., Colburn W.A., DeGruttola V.G., DeMets D.L., Downing G.J., Hoth D.F., Oates J.A., Peck C.C., Schooley R.T., Spilker B.A. (2001). Biomarkers and surrogate endpoints: Preferred definitions and conceptual framework. J. Clin. Pharm. Ther..

[32] Paik S., Shak S., Tang G., Kim C., Baker J., Cronin M., Baehner F.L., Walker M.G., Watson D., Park T. (2004). A multigene assay to predict recurrence of tamoxifen-treated, node-negative breast cancer. N. Engl. J. Med..

[33] Jiang F., Katz R.L. (2002). Use of interphase fluorescence in situ hybridization as a powerful diagnostic tool in cytology. Diagn. Mol. Pathol..

[34] Schena M., Shalon D., Davis R.W., Brown P.O. (1995). Quantitative monitoring of gene expression patterns with a complementary DNA microarray. Science.

[35] Stevens T., Berk M.P., Lopez R., Chung Y.M., Zhang R., Parsi M.A., Bronner M.P., Feldstein A.E. (2012). Lipidomic profiling of serum and pancreatic fluid in chronic pancreatitis. Pancreas.

[36] Anderson N.L. (2002). The human plasma proteome: History, character, and diagnostic prospects. Mol. Cell. Proteomics.

[37] Nilsson T., Mann M., Aebersold R., Yates J.R., Bairoch A., Bergeron J.J. (2010). Mass spectrometry in high-throughput proteomics: Ready for the big time. Nat. Methods.

[38] Kim Y.S., Maruvada P., Milner J.A. (2008). Metabolomics in biomarker discovery: Future uses for cancer prevention. Future Oncol..

[39] MacLellan D.L., Mataija D., Doucette A., Huang W., Langlois C., Trottier G., Burton I.W., Walter J.A., Karakach T.K. (2011). Alterations in urinary metabolites due to unilateral ureteral obstruction in a rodent model. Mol. Biosyst..

[40] Paulo J.A., Urrutia R., Banks P.A., Conwell D.L., Steen H. (2011). Proteomic analysis of an immortalized mouse pancreatic stellate cell line identifies differentially-expressed proteins in activated *vs*. nonproliferating cell states. J. Proteome Res..

[41] Siprashvili Z., Webster D.E., Kretz M., Johnston D., Rinn J.L., Chang H.Y., Khavari P. (2012). Identification of proteins binding coding and non-coding human RNAs using protein microarrays. BMC Genomics.

[42] Van Summeren A., Renes J., Bouwman F.G., Noben J.P., van Delft J.H., Kleinjans J.C., Mariman E.C. (2011). Proteomics investigations of drug-induced hepatotoxicity in HepG2 cells. Toxicol. Sci..

[43] Kalmar A., Wichmann B., Galamb O., Spisák S., Tóth K., Leiszter K., Tulassay Z., Molnár B. (2012). Gene expression analysis of normal and colorectal cancer tissue samples from fresh frozen and matched formalin-fixed, paraffin-embedded (FFPE) specimens after manual and automated RNA isolation. Methods.

[44] Vincenti D.C., Murray G.I. (2012). The proteomics of formalin-fixed wax-embedded tissue. Clin. Biochem..

[45] Teng P., Bateman N.W., Hood B.L., Conrads T.P. (2010). Advances in proximal fluid proteomics for disease biomarker discovery. J. Proteome Res..

[46] Traum A.Z., Wells M.P., Aivado M., Libermann T.A., Ramoni M.F., Schachter A.D. (2006). SELDI-TOF MS of quadruplicate urine and serum samples to evaluate changes related to storage conditions. Proteomics.

[47] Drake S.K., Bowen R.A.R., Remaley A.T., Hortin G.L. (2004). Potential interferences from blood collection tubes in mass spectrometric analyses of serum polypeptides. Clin. Chem..

[48] Hsieh S.Y., Chen R.K., Pan Y.H., Lee H.L. (2006). Systematical evaluation of the effects of sample collection procedures on low-molecular-weight serum/plasma proteome profiling. Proteomics.

[49] Thomas C.E., Sexton W., Benson K., Sutphen R., Koomen J. (2010). Urine collection and processing for protein biomarker discovery and quantification. Cancer Epidemiol. Biomarkers Prev..

[50] Timms J.F., Arslan-Low E., Gentry-Maharaj A., Luo Z., T’Jampens D., Podust V.N., Ford J., Fung E.T., Gammerman A., Jacobs I. (2007). Preanalytic influence of sample handling on SELDI-TOF serum protein profiles. Clin. Chem..

[51] Griffin T.J., Bandhakavi S. (2011). Dynamic range compression: A solution for proteomic biomarker discovery?. Bioanalysis.

[52] Rai A.J., Gelfand C.A., Haywood B.C., Warunek D.J., Yi J., Schuchard M.D., Mehigh R.J., Cockrill S.L., Scott G.B.I., Tammen H. (2005). HUPO Plasma Proteome Project specimen collection and handling: Towards the standardization of parameters for plasma proteome samples. Proteomics.

[53] Sigdel T.K., Kaushal A., Gritsenko M., Norbeck A.D., Qian W.J., Xiao W., Camp D.G., Smith R.D., Sarwal M.M. (2010). Shotgun proteomics identifies proteins specific for acute renal transplant rejection. Proteomics Clin. Appl..

[54] O’Farrell P.H. (1975). High resolution two-dimensional electrophoresis of proteins. J. Biol. Chem..

[55] Shevchenko A., Tomas H., Havlis J., Olsen J.V., Mann M. (2006). In-gel digestion for mass spectrometric characterization of proteins and proteomes. Nat. Protoc..

[56] Martosella J., Zolotarjova N., Liu H., Nicol G., Boyes B.E. (2005). Reversed-phase high-performance liquid chromatographic prefractionation of immunodepleted human serum proteins to enhance mass spectrometry identification of lower-abundant proteins. J. Proteome Res..

[57] Pieper R., Gatlin C.L., McGrath A.M., Makusky A.J., Mondal M., Seonarain M., Field E., Schatz C.R., Estock M.A., Ahmed N. (2004). Characterization of the human urinary proteome: A method for high-resolution display of urinary proteins on two-dimensional electrophoresis gels with a yield of nearly 1400 distinct protein spots. Proteomics.

[58] Smith M.P.W., Wood S.L., Zougman A., Ho J.T.C., Peng J., Jackson D., Cairns D.A., Lewington A.J.P., Selby P.J., Banks R.E. (2011). A systematic analysis of the effects of increasing degrees of serum immunodepletion in terms of depth of coverage and other key aspects in top-down and bottom-up proteomic analyses. Proteomics.

[59] Chen E.I., Hewel J., Felding-Habermann B., Yates J.R. (2006). Large scale protein profiling by combination of protein fractionation and multidimensional protein identification technology (MudPIT). Mol. Cell. Proteomics.

[60] Cairns D.A. (2011). Statistical issues in quality control of proteomic analyses: Good experimental design and planning. Proteomics.

[61] Kentsis A., Lin Y.Y., Kurek K., Calicchio M., Wang Y.Y., Monigatti F., Campagne F., Lee R., Horwitz B., Steen H. (2010). Discovery and validation of urine markers of acute pediatric appendicitis using high-accuracy mass spectrometry. Ann. Emerg. Med..

[62] Cazares L.H., Adam B.L., Ward M.D., Nasim S., Schellhammer P.F., Semmes O.J., Wright G.L. (2002). Normal, benign, preneoplastic, and malignant prostate cells have distinct protein expression profiles resolved by surface enhanced laser desorption/ionization mass spectrometry. Clin. Cancer Res..

[63] Petricoin E.F., Ornstein D.K., Paweletz C.P., Ardekani A., Hackett P.S., Hitt B.A., Velassco A., Trucco C., Wiegand L., Wood K. (2002). Serum proteomic patterns for detection of prostate cancer. J. Natl. Cancer Inst..

[64] Fisher R.A. (1937). The Design of Experiments.

[65] Oberg A.L., Vitek O. (2009). Statistical design of quantitative mass spectrometry-based proteomic experiments. J. Proteome Res..

[66] Sorace J.M., Zhan M. (2003). A data review and re-assessment of ovarian cancer serum proteomic profiling. BMC Bioinform..

[67] Dobbin K., Simon R. (2005). Sample size determination in microarray experiments for class comparison and prognostic classification. Biostatistics.

[68] Ein-Dor L., Zuk O., Domany E. (2006). Thousands of samples are needed to generate a robust gene list for predicting outcome in cancer. Proc. Natl. Acad. Sci. USA.

[69] Molloy M.P., Brzezinski E.E., Hang J., McDowell M.T., VanBogelen R.A. (2003). Overcoming technical variation and biological variation in quantitative proteomics. Proteomics.

[70] Pepe M.S. (2003). The Statistical Evaluation of Medical Tests for Classification and Prediction.

[71] Diz A.P., Truebano M., Skibinski D.O.F. (2009). The consequences of sample pooling in proteomics: An empirical study. Electrophoresis.

[72] Kendziorski C., Irizarry R.A., Chen K.S., Haag J.D., Gould M.N. (2005). On the utility of pooling biological samples in microarray experiments. Proc. Natl. Acad. Sci. USA.

[73] Ibebuogu U.N., Nasir K., Gopal A., Ahmadi N., Mao S.S., Young E., Honoris L., Nuguri V.K., Lee R.S., Usman N. (2009). Comparison of atherosclerotic plaque burden and composition between diabetic and non diabetic patients by non invasive CT angiography. Int. J. Cardiovasc. Imaging.

[74] Burke A.P., Kolodgie F.D., Zieske A., Fowler D.R., Weber D.K., Varghese P.J., Farb A., Virmani R. (2004). Morphologic findings of coronary atherosclerotic plaques in diabetics: A postmortem study. Arterioscler. Thromb. Vasc. Biol..

[75] Qu Y., Adam B.L., Yasui Y., Ward M.D., Cazares L.H., Schellhammer P.F., Feng Z., Semmes O.J., Wright G.L. (2002). Boosted decision tree analysis of surface-enhanced laser desorption/ ionization mass spectral serum profiles discriminates prostate cancer from noncancer patients. Clin. Chem..

[76] Rai A.J., Zhang Z., Rosenzweig J., Shih I.-M., Pham T., Fung E.T., Sokoll L.J., Chan D.W. (2002). Proteomic approaches to tumor marker discovery. Arch. Pathol. Lab. Med..

[77] Mataija-Botelho D., Murphy P., Pinto D.M., Maclellan D.L., Langlois C., Doucette A. (2009). A qualitative proteome investigation of the sediment portion of human urine: Implications in the biomarker discovery process. Proteomics Clin. Appl..

[78] Ambroise C., McLachlan G.J. (2002). Selection bias in gene extraction on the basis of microarray gene-expression data. Proc. Natl. Acad. Sci. USA.

[79] Baggerly K.A., Morris J.S., Wang J., Gold D., Xiao L.C., Coombes K.R. (2003). A comprehensive approach to the analysis of matrix-assisted laser desorption/ionization-time of flight proteomics spectra from serum samples. Proteomics.

[80] Wall M.J., Crowell A.M.J., Simms G.A., Liu F., Doucette A.A. (2011). Implications of partial tryptic digestion in organic-aqueous solvent systems for bottom-up proteome analysis. Anal. Chim. Acta.

[81] Puchades M., Westman A., Blennow K., Davidsson P. (1999). Removal of sodium dodecyl sulfate from protein samples prior to matrix-assisted laser desorption/ionization mass spectrometry. Rapid. Commun. Mass. Spectrom..

[82] Wang N., Xie C., Young J.B., Li L. (2009). Off-line two-dimensional liquid chromatography with maximized sample loading to reversed-phase liquid chromatography-electrospray ionization tandem mass spectrometry for shotgun proteome analysis. Anal. Chem..

[83] Botelho D., Wall M.J., Vieira D.B., Fitzsimmons S., Liu F., Doucette A. (2010). Top-down and bottom-up proteomics of SDS-containing solutions following mass-based separation. J. Proteome Res..

[84] Bellei E., Bergamini S., Monari E., Fantoni L.I., Cuoghi A., Ozben T., Tomasi A. (2011). High-abundance proteins depletion for serum proteomic analysis: Concomitant removal of non-targeted proteins. Amino Acids.

[85] Liu T., Qian W.J., Mottaz H.M., Gritsenko M.A., Norbeck A.D., Moore R.J., Purvine S.O., Camp D.G., Smith R.D. (2006). Evaluation of multiprotein immunoaffinity subtraction for plasma proteomics and candidate biomarker discovery using mass spectrometry. Mol. Cell. Proteomics.

[86] Fernández-Llama P., Khositseth S., Gonzales P.A., Star R.A., Pisitkun T., Knepper M.A. (2010). Tamm-Horsfall protein and urinary exosome isolation. Kidney Int..

[87] Chavez E., Navarro G. (2001). A Probabilistic Spell for the Curse of Dimensionality. Algorithm Eng. Exp..

[88] Bellman R. (1961). Adaptive Control Processes—A Guided Tour.

[89] Pavelka N., Pelizzola M., Vizzardelli C., Capozzoli M., Splendiani A., Granucci F., Ricciardi-Castagnoli P. (2004). A power law global error model for the identification of differentially expressed genes in microarray data. BMC Bioinform..

[90] Choi H., Fermin D., Nesvizhskii A.I. (2008). Significance analysis of spectral count data in label-free shotgun proteomics. Mol. Cell. Proteomics.

[91] Suykens J.A.K., Vandewalle J. (1999). Least squares support vector machine classifiers. Neural Process. Lett..

[92] Wang Y., Lin S., Li H., Kung S. (1998). Data mapping by probabilistic modular networks and information-theoretic criteria. IEEE Trans. Signal Process..

[93] Wang A., Gehan E. (2005). Gene selection for microarray data analysis using principal component analysis. Stat. Med..

[94] Krzanowski W.J. (1987). Selection of variables to preserve multivariate data structure using principal components. J. Roy. Statist. Soc. Ser. C.

[95] Satagopan J.M., Panageas K.S. (2003). A statistical perspective on gene expression data analysis. Stat. Med..

[96] Allison D.B., Cui X., Page G.P., Sabripour M. (2006). Microarray data analysis: From disarray to consolidation and consensus. Nat. Rev. Genet..

[97] Golub T.R., Slonim D.K., Tamayo P., Huard C., Gaasenbeek M., Mesirov J.P., Coller H., Loh M.L., Downing J.R., Caligiuri M.A. (1999). Molecular classification of cancer: Class discovery and class prediction by gene expression monitoring. Science.

[98] Frey B.J., Dueck D. (2007). Clustering by passing messages between data points. Science.

